# Equine bone marrow-derived mesenchymal stromal cells are heterogeneous in MHC class II expression and capable of inciting an immune response *in vitro*

**DOI:** 10.1186/scrt402

**Published:** 2014-01-24

**Authors:** Lauren V Schnabel, Lynn M Pezzanite, Douglas F Antczak, M Julia Bevilaqua Felippe, Lisa A Fortier

**Affiliations:** 1 Department of Clinical Sciences, College of Veterinary Medicine, North Carolina State University, 1060 William Moore Drive, Raleigh, NC 27606, USA; 2 Department of Clinical Sciences, College of Veterinary Medicine, Cornell University, Ithaca, NY 14853, USA; 3 Baker Institute for Animal Health, Cornell University, Micro.Wing, Room 215, Hungerford Hill Road, Ithaca, NY 14853, USA

## Abstract

**Introduction:**

The horse is a valuable species to assess the effect of allogeneic mesenchymal stromal cells (MSCs) in regenerative treatments. No studies to date have examined recipient response to major histocompatibility complex (MHC)-mismatched equine MSCs. The purposes of this study were to immunophenotype MSCs from horses of known MHC haplotype and to compare the immunogenicity of MSCs with differing MHC class II expression.

**Methods:**

MSCs and peripheral blood leukocytes (PBLs) were obtained from Thoroughbred horses (*n* = 10) of known MHC haplotype (ELA-A2, -A3, and -A9 homozygotes). MSCs were cultured through P8; cells from each passage (P2 to P8) were cryopreserved until used. Immunophenotyping of MHC class I and II, CD44, CD29, CD90, LFA-1, and CD45RB was performed by using flow cytometry. Tri-lineage differentiation assays were performed to confirm MSC multipotency. Recombinant equine IFN-γ was used to stimulate MHC class II negative MSCs in culture, after which expression of MHC class II was re-examined. To assess the ability of MHC class II negative or positive MSCs to stimulate an immune response, modified one-way mixed leukocyte reactions (MLRs) were performed by using MHC-matched and mismatched responder PBLs and stimulator PBLs or MSCs. Proliferation of gated CFSE-labeled CD3+ responder T cells was evaluated via CFSE attenuation by using flow cytometry and reported as the number of cells in the proliferating T-cell gate.

**Results:**

MSCs varied widely in MHC class II expression despite being homogenous in terms of “stemness” marker expression and ability to undergo trilineage differentiation. Stimulation of MHC class II negative MSCs with IFN-γ resulted in markedly increased expression of MHC class II. MLR results revealed that MHC-mismatched MHC class II-positive MSCs caused significantly increased responder T-cell proliferation compared with MHC-mismatched MHC class II-negative and MHC-matched MSCs, and equivalent to that of the positive control of MHC-mismatched leukocytes.

**Conclusions:**

The results of this study suggest that MSCs should be confirmed as MHC class II negative before allogeneic application. Additionally, it must be considered that even MHC class II-negative MSCs could upregulate MHC class II expression if implanted into an area of active inflammation, as demonstrated with *in vitro* stimulation with IFN-γ.

## Introduction

The immune status and immunosuppressive properties of adult bone marrow-derived mesenchymal stromal cells (MSCs) have been investigated in multiple species over the past decade with conflicting results [1-4]. Although MSCs are commonly thought of and referred to as immunoprivileged in the literature, multiple studies in both humans and mice have demonstrated that allogeneic adult bone marrow-derived MSCs are capable of eliciting immune responses both *in vitro* and *in vivo*[1,5-9]. In these studies, the immunosuppressive effects of MSCs were unable to prevent an immunogenic response *in vitro*, or to prevent MSC rejection *in vivo*. Complicating our understanding of the immune status of MSCs is the fact that not all MSCs described in the literature have the same major histocompatibility complex (MHC) class II expression profile [5,6,10], and some studies did not include MSC immunophenotyping and/or proper experimental controls [1].

Adult mesenchymal stem cells are increasingly used in regenerative therapies for equine patients [11-16]. In cases in which treatment is indicated at the time of diagnosis, the use of banked allogeneic MSCs would be advantageous instead of having to wait several weeks to culture autologous MSCs. Bone marrow-derived MSCs isolated from young-adult horses have been previously phenotyped during mid-late passage (P3 to P7) as MHC class II negative [17-19], and equine allogeneic MSCs have been reported to be both immunoprivileged and immunosuppressive *in vitro*[19], as well as nonimmunogenic *in vivo*[20]. No studies have examined the immunophenotype of equine MCSs isolated from horses of varying ages or sequentially over early to late passages. In addition, no studies to date have used MSCs and leukocytes isolated from horses of known MHC haplotype, which is essential for performing MHC-matched and MHC-mismatched studies. As the horse is a valuable species for assessing the effect of MSC treatment on musculoskeletal disorders such as tendonitis, cartilage damage, and osteoarthritis [13,21-26], it is critical to understand the immune status of equine MSCs before evaluating the use of allogeneic MSCs for “off the shelf” therapy in such models.

The purposes of this study were (a) to phenotype P2-P8 bone marrow-derived MSCs from horses of known MHC haplotypes; and (b) to compare the immunogenicity of MSCs with differing immunophenotypes, particularly in regard to MHC class II expression, through modified one-way mixed leukocyte reactions (MLRs). This is the first equine study to evaluate the immune response elicited by MHC-matched and MHC-mismatched MSCs, including controls of MHC-matched and -mismatched peripheral blood leukocytes (PBLs). Our first hypothesis, based on previous human early-passage MSC immunophenotyping [10], was that early-passage equine MSCs would be heterogeneous in MHC class II expression. Our second hypothesis was that MHC-mismatched MHC class II-negative MSCs would have low immunogenicity *in vitro*, whereas those that were positive would be immunogenic.

## Methods

### Horses

Thoroughbred horses of known MHC haplotype belonging to the Equine Genetics Center at the James A. Baker Institute for Animal Health at Cornell University were used in these studies. All horses were MHC homozygotes of equine leukocyte antigen (ELA) haplotypes ELA-A2, ELA-A3, or ELA-A9, as previously determined by ELA serotyping, direct MHC gene sequencing, and microsatellite typing [27-30]. The Institutional Animal Care and Use Committee of Cornell University approved the use of horses in these studies.

### Peripheral blood leukocytes

Blood was collected via jugular venipuncture with extension sets (Baxter Healthcare, Deerfield, IL, USA) and 16-gauge needles into 500-ml evacuated containers (Baxter Healthcare, Deerfield, IL, USA) containing 7,500 units of heparin (Sigma-Aldrich, St. Louis, MO, USA) each. Plasma was allowed to separate for 20 minutes at room temperature, and peripheral blood leukocytes (PBLs) were then isolated from the plasma via carbonyl iron (Sigma-Aldrich) granulocyte depletion and Ficoll-Paque Plus (Amersham Biosciences, Piscataway, NJ, USA) gradient centrifugation [31]. PBLs were resuspended in RPMI 1640 medium (Gibco, Grand Island, NY, USA) containing 10% fetal bovine serum (FBS), 0.1 m*M* 2-mercaptoethanol, penicillin (100 units/ml), and streptomycin (100 μg/ml), and fresh cells were used for all experiments.

### Dermal fibroblasts

For dermal fibroblast isolation, 6-mm dermal punch biopsies were collected aseptically from the neck under standing sedation with local anesthesia and placed into a 100-mm tissue-culture dish containing phosphate-buffered saline (PBS) with penicillin (100 units/ml) and streptomycin (100 μg/ml). The biopsies were then individually rinsed with 70% ethanol, quickly passed through the flame of a Bunsen burner, and placed into to a new 100-mm tissue-culture dish containing PBS with penicillin and streptomycin. The epidermis was then sharply dissected from the dermis on each biopsy by using a number 10 scalpel blade, and discarded. The dermal biopsies were digested overnight in a spinner flask at 37°C with collagenase IV (Life Technologies, Carlsbad, CA, USA) at a concentration of 7,500 units/gram tissue diluted in dermal fibroblast (DF) media consisting of high glucose (4 g/dl) DMEM media (Gibco) containing 10% FBS, penicillin (100 units/ml), and streptomycin (100 μg/ml) at a volume of 5 ml/g of tissue. After digestion, the cell suspension was passed through a 100-μm cell strainer, pelleted, washed with PBS, and then plated onto 175 cm^2^ tissue-culture flasks at a density of 1 × 10^4^ cells/cm^2^ in DF media. The DFs were culture expanded to P2. Cells to be aliquoted and cryopreserved for flow cytometry were pelleted after dissociation, resuspended in freeze media (DF media with 10% FBS and 10% dimethyl sulfoxide), and frozen at 5 × 10^6^ cells/cryovial.

### Bone marrow aspirate collection and isolation of MSCs

Bone marrow aspirate was collected aseptically from the sternum of 10 horses by using 11-gauge Jamshidi bone marrow biopsy needles under standing sedation with local anesthesia. For each harvest, a total of 120 ml of aspirate was collected into 60-ml syringes containing 25,000 units of heparin each. Three horses underwent a second aspirate collection 2 months after the first, for a total of 13 aspirates (six ELA-A2, six ELA-A3, one ELA-A9 haplotypes). Bone marrow aspirates were purified via Ficoll-Paque Plus (Amersham Biosciences) gradient centrifugation, as previously described [32] and plated onto 100-mm tissue-culture plates in low glucose (1 g/dl) DMEM media (Gibco) containing 10% FBS, 2 m*M* l-glutamine, penicillin (100 units/ml), streptomycin (100 μg/ml), and basic fibroblastic growth factor (bFGF, 1 ng/ml).

MSCs were expanded over one passage, such that cryopreserved passage 2 (P2) stocks were obtained for each aspirate. P2 MSCs were later thawed and cultured through P8 to examine potential MHC class II expression changes over time and to compare MHC class II expression with that previously described for mid- to late-passage cells [17-19]. At each passage, MSC stocks were cryopreserved for immunophenotyping and mixed leukocyte reactions (MLRs). Throughout culturing, media were exchanged every 48 hours. Cells were passaged 1:3 at approximately 80% subconfluency by using Accumax cell-dissociation solution (Innovative Cell Technologies, Inc., San Diego, CA, USA) and plated at a density of approximately 1 × 10^4^ cells/cm^2^. Cells to be cryopreserved were pelleted after dissociation, resuspended in freeze media (MSC media as described earlier with 10% FBS and 10% dimethyl sulfoxide), and frozen at either 5 × 10^6^ cells/cryovial for expansion and flow cytometry or 1 × 10^6^ cells/cryovial for use in MLR experiments [21].

### Immunophenotyping of MSCs

MSCs were immunophenotyped at each passage (two to eight) for expression levels of MHC class I, MHC class II, and a panel of positive (CD44, CD29, CD90) and negative (CD11a/CD18, CD45RB) markers by using flow cytometry. Antibodies for these markers were previously validated for the horse and described by our laboratory [32]. Dilutions of 1:200 (CD29, CD90), 1:100 (CD44), or 1:10 (MHC class I, MHC class II, CD11a/CD18, CD45RB) were used, according to the manufacturer’s directions for commercial antibodies and according to previous experience for antibodies produced in the Antczak Laboratory [32]. PBLs and P2 DFs were used as controls. MSCs from each horse were directly compared with their own PBLs for expression levels of MHC class II to ensure that any variability in MSC MHC class II expression was not due to individual horse variability in MHC class II antibody binding.

Cells were pelleted in aliquots containing approximately 1 × 10^6^ cells on 96-well V-bottom plates and treated with a 20-minute blocking step by using 10% normal goat serum in phosphate-buffered saline (PBS). The cells were pelleted and resuspended in unconjugated primary monoclonal antibody and incubated for 45 minutes at 4°C. Cells were then washed, a secondary fluorescent-conjugated goat anti-mouse IgG antibody (fluorescein isothiocyanate (FITC; read on FL1) or allophycocyanin (APC; read on FL4); BD Biosciences, San Jose, CA, USA) was applied to the unconjugated antibodies, and the samples incubated for an additional 45 minutes at 4°C. Cells were washed and then resuspended in PBS and analyzed on a FACSCalibur (Becton Dickinson Immunocytometry Systems) flow cytometer equipped with 488-μm argon and 635-μm red diode lasers and BD Cell Quest analysis software (BD Biosciences). Cells exposed to mouse antiparvovirus antibody and FITC or APC-conjugated secondary antibodies were used as negative isotype controls.

Cells were gated as previously determined for cultured MSCs by our laboratory [32], and data were collected on 2 × 10^4^ cells for each sample.

### MSC differentiation assays

To verify that the MSCs were capable of tri-lineage differentiation, 5 × 10^6^ P3 cells were used for adipogenic, osteogenic, and chondrogenic induction assays [32]. Induced P3 DFs were used as a control, in addition to noninduced P3 MSCs.

Adipogenic induction was performed by using the commercially available STEMPRO® Adipogenesis Differentiation Kit (Gibco) according to the manufacturer’s instructions. Media was exchanged every 3 to 4 days until termination of induction on day 14. At that time, cells were fixed in 4% paraformaldehyde, stained with Oil-Red-O for identification of lipid inclusions, and counterstained with hematoxylin. Stained cells were imaged with standard microscopy and assessed for Oil-Red-O staining.

Osteogenic induction was performed by using the commercially available STEMPRO® Osteogenesis Differentiation Kit (Gibco) according to the manufacturer’s instructions. Media was exchanged every 3 to 4 days until termination of induction on day 14, at which time the cells were fixed in 4% paraformaldehyde, stained with 2% aqueous Alizarin Red for identification of calcium deposits, and counterstained with hematoxylin. Stained cells were imaged with standard microscopy and assessed for Alizarin Red staining.

Chondrogenic induction was performed on pellet cultures containing 5 × 10^5^ cells/pellet [33] by using the commercially available STEMPRO Chondrogenesis Differentiation Kit (Gibco) according to the manufacturer’s instructions. Media was exchanged every 3 to 4 days until termination of induction on days 14 and 28. Pellets were fixed in 4% paraformaldehyde, embedded in paraffin, and sectioned (4 μm). Sections were stained with Safranin-O/fast green and Alcian blue, counterstained with hematoxylin, and assessed for matrix metachromasia by using standard microscopy.

### IFN-ɣ stimulation of MSCs

To determine whether equine MSCs were capable of upregulating their MHC class II expression, recombinant equine IFN-γ (R&D Systems Inc., Minneapolis, MN, USA) was used to stimulate MSCs in culture, after which expression levels of MHC class II were reassessed with flow cytometry. MSCs were plated on 100-mm tissue-culture plates at a density of 1 × 10^4^ cells/cm^2^ in MSC media, as described earlier, and allowed to adhere to plates. At 24 hours in culture, media on control plates were exchanged with fresh MSC media, whereas media on treated plates were exchanged with fresh MSC media containing 100 ng/ml of IFN-γ [17,34,35]. At 72 hours, media on control and treated plates were again exchanged in the same manner. At 96 hours, cells were dissociated from the plates and analyzed for MHC class II expression by using flow cytometry, as described earlier for immunophenotyping.

### MHC class II antibody comparison

Because previously published studies examining MHC class II expression levels of equine MSCs with flow cytometry mostly used a commercially available MHC class II antibody (clone CVS20; AbD Serotec, Raleigh, NC, USA) [19], we compared this antibody with the one used in this study for immunophenotyping (cz11, clone 130.8E8D9, Laboratory of Dr. D. Antczak, Cornell University, Ithaca, NY, USA). Both antibodies were generated from mouse hybridomas and are of the IgG1 isotype. Clone CVS20 was used at a dilution of 1:100, as recommended by the manufacturer, and cz11, clone 130.8E8D9 was used at a dilution of 1:10, as stated earlier. PBLs, MSCs, and MSCs stimulated with IFN-γ were used to make comparisons in MHC class II expression levels by using the two antibodies. For both primary mouse anti-horse MHC class II antibodies, a secondary goat anti-mouse APC antibody (BD Biosciences) was used. Cells exposed to mouse antiparvovirus antibody with the same secondary antibody were used as negative isotype controls.

### Modified one-way mixed leukocyte reactions

To assess the ability of MSCs to stimulate an immune response, modified one-way mixed leukocyte reactions (MLRs) were performed in duplicate on 24-well tissue-culture plates by using MHC-matched and mismatched responder PBLs and stimulator MSCs. MHC-matched stimulator PBLs were used as negative MLR controls (baseline T-cell proliferation), and MHC-mismatched stimulator PBLs were used as positive MLR controls. Responder PBLs were labeled with 5(6)-carboxyfluorescein diacetate *N*-succinimidyl ester (0.25 μg/ml of cell solution, CFSE, Sigma-Aldrich and examined at two different concentrations (1.5 × 10^6^ and 2.5 × 10^6^ cells/well). Proliferative ability of responder cells was verified via mitogen stimulation with concanavalin A (ConA, 5 μg/ml; Sigma-Aldrich). Stimulator MSCs were plated at 5 × 10^4^ cells/well in MSC media 24 hours before the addition of responder PBLs, such that MSCs would be approximately 80% confluent by the end of the experiment. Stimulator PBLs were irradiated with 9 Gy from a Cs-137 source to inhibit proliferation and plated at 1.2 × 10^6^ cells/well immediately before the addition of responder PBLs.

The resultant ratios of responder-to-stimulator PBLs was based on previously published equine MLR experiments [31] and determined to be optimal for these studies in preliminary experiments. Cultures were maintained for 5 days with modified RPMI 1640 media (1 ml/well) containing 10% FBS, 0.1 m*M* 2-mercaptoethanol, penicillin (100 units/ml), streptomycin (100 μg/ml), and basic fibroblastic growth factor (bFGF, 1 ng/ml). Media were not exchanged over the 5 days. After culture, PBLs were aspirated from the wells and stained with a primary mouse anti-horse CD3 antibody (clone UC F6G-3.3; Laboratory of Dr. J. Scott, University of California Davis, Davis, CA, USA) and a secondary goat anti-mouse APC antibody (BD Biosciences). The antibody-staining process for flow-cytometry analysis was performed as described earlier for immunophenotyping.

Proliferation of gated CFSE-labeled CD3-positive responder T cells was evaluated via CFSE attenuation by using flow cytometry. Cells were first gated on FL4 so that only the CD3-positive cells (T cells) were then examined on FL1 for CFSE attenuation. Nonstimulated responder T cells were used to set the boundary of nonproliferating cells, such that all cells to the left (lower fluorescence intensity on FL1) of that boundary were determined to be proliferating. The number of cell counts in the proliferating T-cell gate was measured, as well as the CFSE Geometric Mean Fluorescence Intensity (GMFI) of all T cells, to reflect the extent of proliferation. Data were collected on the entirety of each sample because cell numbers were being measured.

MLRs were performed in a total of five experiments by using responder PBLs from four different horses (two ELA-A2, one ELA-A3, and one ELA-A9 haplotypes) and stimulator PBLs from three different horses (one ELA-A2 and two ELA-A3 haplotypes). In each experiment, T-cell proliferation in response to MHC-matched MSCs, MHC-mismatched MHC class II-negative MSCs, and MHC-mismatched MHC class II-positive MSCs was assessed. As stated, T-cell proliferation in response to MHC-matched PBLs (MHC-matched MLR) was used as a negative control and set as the baseline proliferation value, whereas T-cell proliferation in response to MHC-mismatched PBLs (MHC-mismatched MLR) was used as a positive control. Because of naturally occurring variation in PBL responses between horses and experiments, the relative T-cell proliferation and relative GMFI in each experiment was reported as the fold change from that of the MHC-matched MLR.

### Statistical analyses

Immunophenotyping data were analyzed with Pearsons correlations. MHC class II expression data obtained by the two different antibodies were analyzed with paired *t* tests. MLR data were normalized by log transformation and analyzed with analysis of covariance (ANCOVA), with horse as a covariate, followed by the Tukey test for multiple comparisons. All analyses were performed by using Statistix 9 software (Analytical Software, Tallahassee, FL, USA), and significance was set at *P* < 0.05.

## Results

### MSC isolation and immunophenotyping

**Figure 1:**
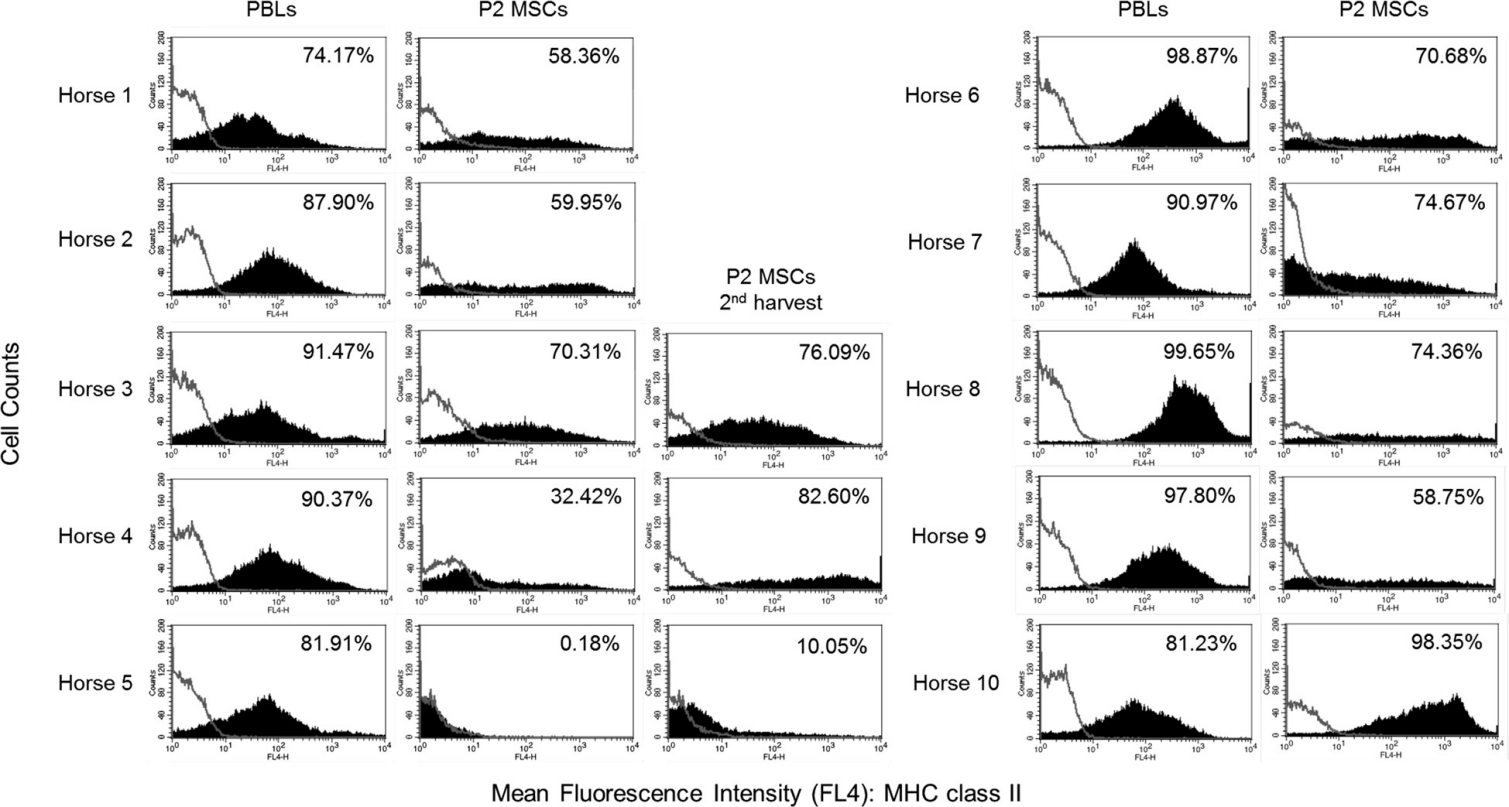
**Flow-cytometric histogram analyses of MHC class II expression in peripheral blood leukocytes (PBLs) and passage 2 bone marrow-derived mesenchymal stromal cells (P2 MSCs) for horses 1 through 10.** The open lines represent negative isotype control staining, and the shaded curves represent MHC class II staining. The percentage of positive cells is in the upper right-hand corner of each histogram. Note the relatively minor variation in PBL MHC class II expression between horses as compared with the major variation in P2 MSC MHC class II expression.

MSCs were isolated and expanded from 13 of 13 bone marrow aspirates. The percentage of P2 MSCs positive for MHC class II expression varied widely among horses, despite fairly consistent results for PBL MHC class II expression, indicating that the MSC variation observed was not due to differences in antibody binding (Figure 1). P2 MSCs from 11 of the 13 aspirates were positive for MHC class II expression with broad and diffuse expression peaks, as opposed to a well-defined narrow peak or two or more peaks suggestive of different but limited cell populations (Figure 1). P2 MSCs from only one horse (horse 5, ELA-A3 haplotype) were negative for MHC class II expression. P2 MSCs from all 13 aspirates had the previously described equine MSC phenotype of MHC I^hi^, CD44^hi^, CD29^hi^, and CD45RB^lo^ (Table 1) [32]. Also consistent with previous studies between 17 and 21 days of culture, variable expression of CD90 and CD11a/CD18 surface molecules [32] was seen, with the majority of the P2 MSCs having a phenotype of CD90^hi^ and CD11a/CD18^lo^ (Table 1). It is important to note that P2 DFs shared a very similar phenotype when examined by using these markers (Table 1) but were consistently MHC class II^lo^ or negative.

**Table 1:** Percentage of equine mesenchymal stromal cells (MSCs) and dermal fibroblasts (DFs) positive for expression of cell-surface markers

	***Percentage of cells positive***						
***P2 MSCs: horse (ELA haplotype; age)***	**MHC I**	**MCH II**	**CD44**	**CD29**	**CD90**	**LFA-1**	**CD45RB**
1 (A3; 20 years)	98.77	58.36	75.94	91.59	30.35	56.05	1.34
2 (A2; 12 years)	98.01	59.95	92.92	97.26	66.34	11.43	0.46
3 (A3; 3 years)	98.67	70.31	90.80	96.86	64.60	15.64	0.57
3 (A3; 3 years) 2nd harvest	95.94	76.09	84.11	97.47	61.48	28.69	1.43
4 (A2; 3 years)	96.51	32.42	78.67	94.42	85.94	0.72	0.41
4 (A2; 3 years) 2nd harvest	98.16	82.61	85.84	96.45	88.50	8.20	3.27
5 (A3; 6 years)	98.20	0.18	95.59	97.68	94.37	0.30	1.58
5 (A3; 6 years) 2nd harvest	98.13	10.05	91.54	97.39	92.25	4.31	5.44
6 (A2; 5 years)	98.68	70.68	93.07	97.27	86.41	0.75	0.98
7 (A3; 19 years)	99.07	74.67	92.62	98.34	55.05	20.92	2.44
8 (A9; 11 years)	98.17	74.36	92.09	97.06	92.27	2.92	1.17
9 (A2; 6 years)	99.22	58.75	96.39	98.93	96.39	0.79	2.95
10 (A2; 5 years)	99.57	98.35	95.57	99.05	50.51	43.49	1.38
*P2 DFs*	97.42	5.53	93.50	96.83	95.09	4.66	4.64

Variability was observed in P2 MSC morphology, in that some MHC class II-positive MSCs had the classic long spindle-shaped morphology equivalent to that of the MHC class II-negative MSCs, whereas other MHC class II-positive MSCs were more triangular to polygonal and smaller (see Additional file 1: Figure S1). All MSCs isolated from older horses (≥10 years of age) displayed some degree of atypical morphology.

MSCs from six of the 11 aspirates positive for MHC class II expression at P2 remained positive through P8, whereas MSCs from the other five aspirates became negative over time in culture, generally by P4 or P5 (Table 2). Later-passage MHC class II-positive MSCs either maintained a broad and diffuse expression peak or converted to a narrower peak, indicating upregulation of MHC class II in some cases (see Additional file 2: Figure S2). P2 MSCs negative for MHC class II expression (two aspirates from horse 5, ELA-A3 haplotype) remained negative through P8. MSCs from all 13 aspirates each maintained their morphology exhibited in P2, regardless of whether they converted from MHC class II positive to MHC class II negative. Only in this single ELA-A3 haplotype horse with MHC class II-negative MSCs from P2-P8 could a correlation be made between MHC haplotype and MSC MHC class II expression. Interestingly, of the MSCs isolated from the four older horses in this study ≥10 years of age (2 ELA-A3, 1 ELA-A2, and 1 ELA-A9 haplotypes), all were strongly positive for MHC class II at P2, and only those of the ELA-A9 haplotype became negative later in the culture period at P7 and P8 (Table 2).

**Table 2:** Percentage of equine mesenchymal stromal cells (MSCs) positive for expression of MHC class II over multiple passages in culture

	***Percentage of cell positive for MHC class II***						
***MSCs: horse (ELA haplotype; age)***	**P2**	**P3**	**P4**	**P5**	**P6**	**P7**	**P8**
1 (A3; 20 years)	58.36	54.67	63.07	53.54	46.22	51.72	38.44
2 (A2; 12 years)	59.95	78.58	98.14	97.25	91.45	94.47	97.62
3 (A3; 3 years)	70.31	1.53	1.35	1.75	0.86	0.85	0.68
3 (A3; 3 years) 2nd harvest	76.09	65.22	59.74	64.83	50.49	48.56	45.14
4 (A2; 3 years)	32.42	8.19	4.03	4.52	2.56	0.49	0.50
4 (A2; 3 years) 2nd harvest	82.60	59.25	56.05	65.31	64.85	67.08	68.96
5 (A3; 6 years)	0.18	0.57	0.29	0.77	0.44	0.08	1.38
5 (A3; 6 years) 2nd harvest	10.05	1.05	0.90	1.59	0.47	0.51	0.45
6 (A2; 5 years)	70.68	72.32	64.62	22.10	2.32	1.93	1.29
7 (A3; 19 years)	74.67	66.49	93.13	94.54	93.68	93.33	95.43
8 (A9; 11 years)	74.36	71.32	67.04	41.82	25.97	8.16	0.90
9 (A2; 6 years)	58.75	41.79	4.92	0.78	0.76	0.48	0.46
10 (A2; 5 years)	98.35	91.92	95.69	96.15	94.89	91.39	85.01

Over the culture period through P8, all MSCs maintained the phenotype of MHC I^hi^, CD44^hi^, CD29^hi^, and CD45RB^lo^, as well as the previously observed variability in expression of CD90 and CD11a/CD18 surface molecules. The median percentage of cells positive for CD90 was 89.75% (range, 3.58% to 99.64%), and the median percentage of cells positive for CD11a/CD18 was 1.53% (range, 0.12% to 70.85%). A weak but significant negative correlation was found between CD90 and MHC class II expression (*r* = −0.23; *P* = 0.03), and a moderate positive correlation was found between CD11a/CD18 and MHC class II expression (*r* = 0.40; *P* < 0.01).

### MSC differentiation assays

**Figure 2:**
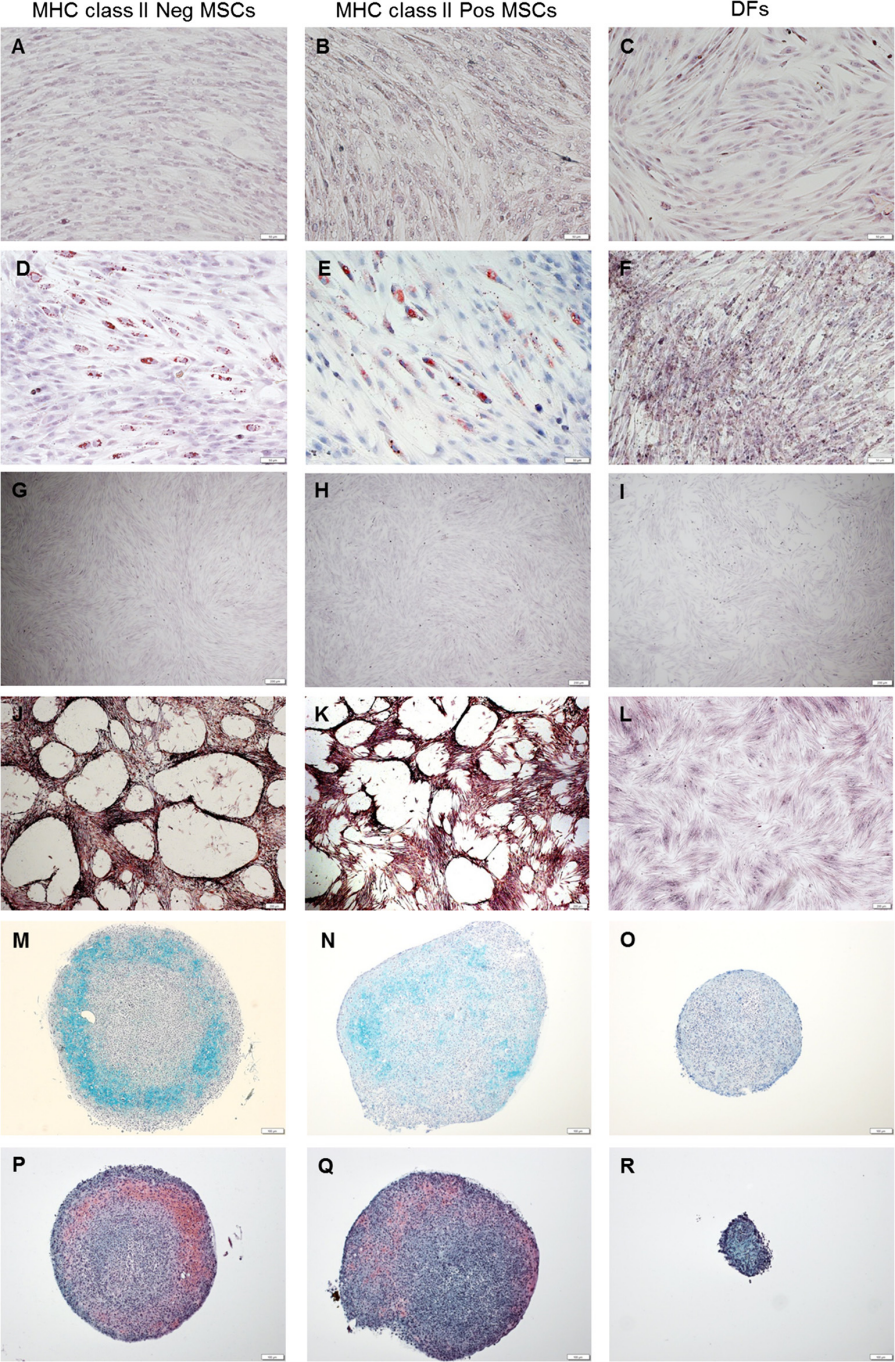
**Trilineage differentiation assay results for MHC class II-negative MSCs, MHC class II-positive MSCs, and dermal fibroblasts (DFs). (A-C)** control Oil-Red-O staining of noninduced cell types; bar = 50 μm. **(D-F)** Oil-Red-O staining after 14 days of adipogenic induction; bar = 50 μm. **(G-I)** control Alizarin Red staining of noninduced cell types; bar = 200 μm. **(J-L)** Alizarin Red staining after 14 days of osteogenic induction; bar = 200 μm. **(M-O)** Alcian blue staining of pellet cultures after 14 days of chondrogenic differentiation; bar = 100 μm. **(P-R)** Safranin-O/fast green staining of pellet cultures after 14 days of chondrogenic differentiation; bar = 100 μm. Both MHC class II-negative and MHC class II-positive MSCs were able to undergo trilineage differentiation, whereas DFs were not.

Trilineage differentiation capacity of both MHC class II negative and MHC class II positive MSCs was confirmed through *in vitro* adipogenic, osteogenic, and chondrogenic induction assays (Figure 2). DFs failed to undergo trilineage differentiation by using the same induction assays, as determined by lack of staining and lack of proper induced cellular morphology (Figure 2).

### IFN-γ stimulation of MSCs and MHC class II antibody comparison

**Figure 3:**
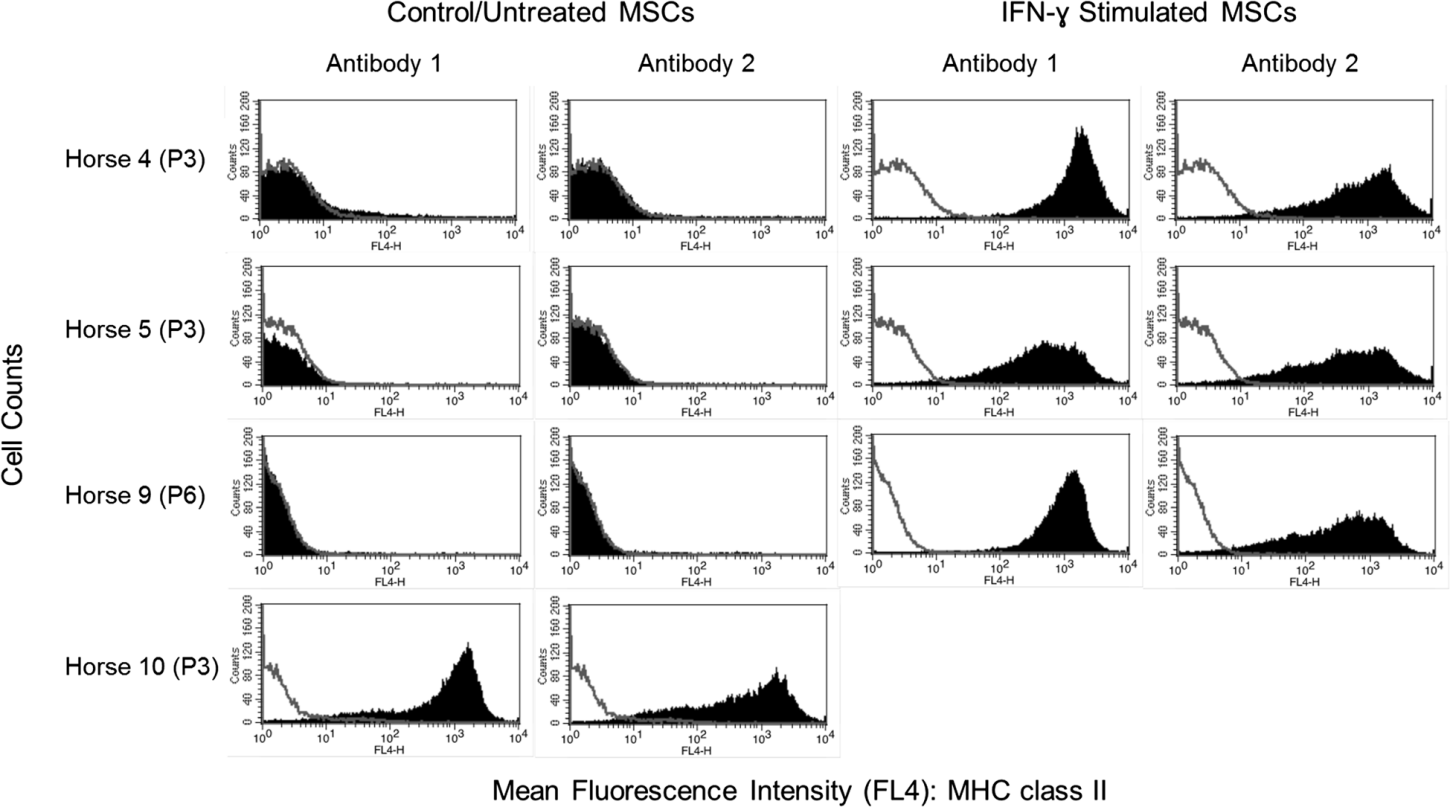
**Flow-cytometric histogram analyses of MHC class II expression in control or untreated bone marrow-derived stromal cells (MSCs) and in IFN-γ-stimulated MSCs used to compare the antibody used in this study (Antibody 1) with the commercially available antibody (Antibody 2).** The open lines represent negative isotype control staining, and the shaded curves represent MHC class II staining. Note the consistency observed between antibodies for all cell types, with the exception of the fact that MSC class II-positive MSCs generally displayed more diffuse fluorescence intensity when stained with Antibody 2 compared with Antibody 1.

Stimulation of MHC class II negative MSCs with IFN-γ resulted in markedly increased expression of MHC II (Figure 3) in both the percentage of cells positive for MHC class II and the fluorescence intensity of the cells. No significant differences were found between MHC class II expression levels (percentage of cells positive) obtained by the two different antibodies for PBLs (*n* = 3; *P* = 0.83), MSCs (*n* = 4; *P* = 0.41), or IFN*-*γ-stimulated MSCs (*n* = 3; *P* = 0.20). Some variability was observed, however, in the fluorescence intensity of the MSCs stained with the different antibodies, with the commercially available antibody (Antibody 2, clone CVS20) generating a more diffuse intensity than the antibody used in this study (Antibody 1, cz11, clone 130.8E8D9) (Figure 3).

### Modified one-way mixed leukocyte reactions

**Figure 4:**
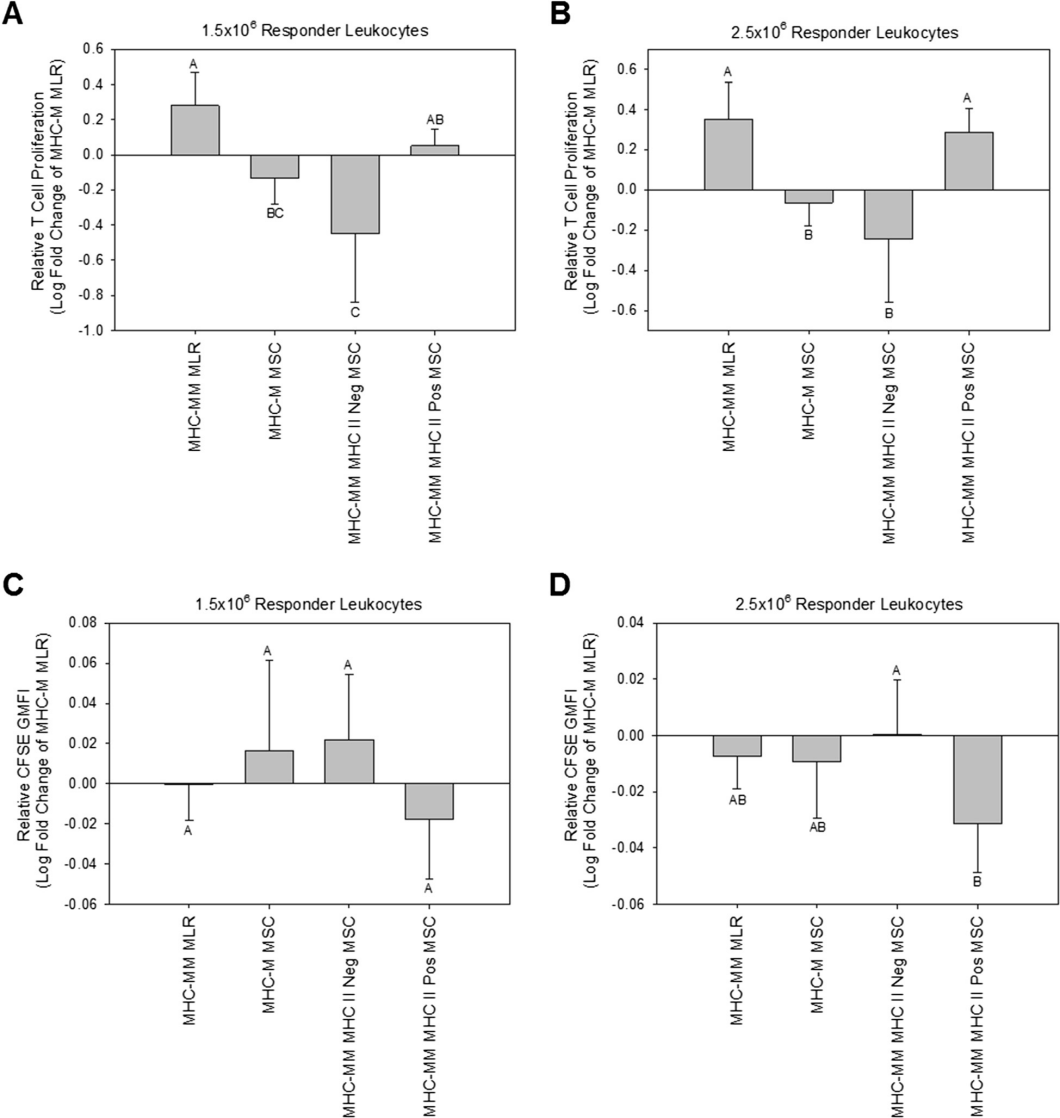
**Modified one-way mixed leukocyte reaction (MLR) results at the lower responder cell concentration of 1.5 × 10**^**6 **^**leukocytes (A, C) and at the higher responder cell concentration of 2.5 × 10**^**6 **^**leukocytes (B, D), as measured by relative responder T-cell proliferation (A, B) and CFSE geometric mean fluorescence intensity (GMFI; C, D).** Bars represent mean ± SD of *n* = 5. Superscript letters indicate significant differences between groups by ANCOVA, with horse as a covariate, followed by the Tukey test for multiple comparisons, *P* < 0.05. MHC-M, MHC-matched; MHC-MM, MHC-mismatched. MHC-mismatched MHC class II-negative MSCs caused significantly less responder T-cell proliferation compared with both the positive control of MHC-mismatched PBLs (MHC-MM MLR) and MHC-mismatched MHC class II-positive MSCs at both responder leukocyte concentrations **(A, B)**. MHC-mismatched MHC class II-negative MSCs resulted in a significantly greater CFSE GMFI, indicative of fewer responder T-cell divisions, compared with MHC-mismatched MHC class II-positive cells at the higher responder leukocyte concentration **(D)**.

MHC-mismatched MHC class II-positive MSCs caused a significant increase in responder T-cell proliferation compared with MHC-mismatched MHC class II-negative MSCs at the lower responder PBL concentration of 1.5 × 10^6^ cells (Figure 4A; *P* < 0.01) and compared with both MHC-mismatched MHC class II-negative and MHC-matched MSCs at the higher responder PBL concentration of 2.5 × 10^6^ cells (Figure 4B; *P* < 0.01). At both responder T-cell concentrations, proliferation caused by MHC-mismatched MHC class II-positive MSCs was statistically equivalent to that caused by the positive control of MHC-mismatched PBLs. MHC-mismatched MHC class II-negative MSCs used in experiments were Horse 5 (ELA-A3) P2 MSCs and Horse 9 (ELA-A2) P5 MSCs. MHC-mismatched MHC class II-positive MSCs used in experiments were Horse 7 (ELA-A3) P2 MSCs and Horse 5 (ELA-A3) P2 IFN-γ-stimulated MSCs. Responder T-cell proliferation results for individual experiments are shown in Additional file 3: Figure S3.

Stimulation of responder PBLs with MHC-mismatched MHC class II-positive MSCs resulted in lower responder T-cell GMFI, indicative of an increased number of cell divisions, compared with MHC-mismatched MHC class II-negative MSCs (Figure 4C, D). This result was significant at the higher responder PBL concentration of 2.5 × 10^6^ cells (Figure 4D; *P* = 0.02). At both responder PBL concentrations, stimulation of responder PBLs with either MHC-mismatched MHC class II-negative MSCs or MHC-mismatched MHC class II-positive MSCs resulted in responder T-cell GMFI statistically equivalent to that of both MHC-matched MSCs and MHC-mismatched PBLs.

## Discussion

This study reports the heterogeneous MHC class II immunophenotype of bone marrow-derived MSCs isolated from horses of varying ages and MHC haplotypes. The MSCs were otherwise homogenous in terms of size and granularity on flow cytometry (see Additional file 4: Figure S4), traditional MSC “stemness” marker profile, and ability to undergo trilineage differentiation. We hypothesized that MSCs would variably express MHC class II during early passages, as was previously shown for human MSCs [10], and that this expression would decrease over time in culture, such that MSCs would be negative for MHC class II expression at later passages. Although this turned out to be the case for MSCs isolated from certain horses, others remained strongly positive for MHC class II expression through P8. This variability observed in MHC class II expression was not due to differences in antibody binding between horses, as determined by comparison of PBL MHC class II expression and also was not due to differences in antibody binding between the antibody primarily used in this study (Antibody 1) and the antibody used in previous studies (Antibody 2), as demonstrated in Figure 3. Interestingly, even MSCs isolated from different bone marrow aspirate harvests from the same horse were found to differ in MHC class II expression.

These results suggest that a combination of factors including genetics, bone marrow aspirate quality, immunologic background at a given time, and culture conditions are responsible for the extreme heterogeneity of MHC class II expression observed. It is possible that the bone marrow origin of these MSCs enabled such heterogeneity, but a direct comparison to adult MSCs of a different origin such as adipose tissue was not performed, and therefore, a conclusion on this matter cannot be made.

As for the majority of the equine MSCs in this study, human and mouse MSCs found to be MHC class II positive displayed a diffuse or broad MHC class II expression peak on flow cytometry histogram analysis, suggestive that the individual MSCs themselves varied in terms of the number of MHC class II molecules expressed on their cell surfaces [5,6]. Both human and mouse MHC class II MSCs in these previous studies otherwise had the expected profile of positive and negative markers for “stemness” and were capable of trilineage differentiation as typical for MSCs. Similarly, MHC class II-positive equine MSCs in this study remained as one homogeneous population within the previously determined MSC gate and consistently expressed the CD44^hi^, CD29^hi^, and CD45RB^lo^ phenotype [32].

At no time during flow-cytometric analysis of MSCs from any horse did there appear to be two or more distinct cell types or cells with distinct marker profiles within the MSC gate (Additional file 4: Figure S4). MHC class II-positive equine MSCs in this study were capable of *in vitro* adipogenic, osteogenic, and chondrogenic differentiation. These findings suggest that equine MSCs themselves are capable of extreme variation in MHC class II expression. Because of the inability to distinguish equine MSCs from DFs based on the “stemness” marker profile alone, the tri-lineage differentiation assays were critical in this study to prove that MHC class II-negative and MHC class II-positive MSCs were multipotent cells and that they had not differentiated into fibroblasts. To the authors’ knowledge, a direct comparison between MSCs and DFs in terms of their ability to undergo trilineage differentiation has not previously been performed.

The variation observed in expression of the cell-surface markers CD90 and CD11a/CD18 (LFA-1) is difficult to interpret, as it was previously shown that equine bone marrow-derived MSCs express variable levels of these markers, and that their expression changes over time in culture, such that cells are CD90^lo^ and CD11a/CD18^hi^ early on, but become CD90^hi^ and CD11a/CD18^hi^ over time [32]. Such variability may be expected when examining cells from different donors. A weak negative correlation was found between CD90 and MHC class II expression when analyzing the phenotype data as a whole, but this correlation was not consistent for the same MSCs over multiple passages. Numerous examples of MSCs remained strongly positive for MHC class II over several passages, but expression of CD90 increased over those same passages. Similarly, although a moderate positive correlation was found between CD11a/CD18 and MHC class II expression, numerous examples of MSCs remained MHC class II positive over several passages but whose expression of CD11a/CD18 decreased. These findings suggest that such correlations may be a more temporal finding than a defining finding, as the greatest numbers of MSCs were MHC class II positive during early passages, when we would expect CD90 expression to be low and CD11a/CD18 expression to be high [32].

The finding that the majority of passage 2 through 4 MSCs in this study were positive for MHC class II expression has not been previously described. Equine studies examining MHC class II expression and immunogenic properties of MSCs to date have largely focused on later passage cells (P3 or later) [17-19], even though most MSCs used in experimental models and in clinical applications are early passage (P2) to maintain proliferative ability [21,22,36,37]. This knowledge is critical for potential allogeneic applications, as MHC-mismatched MHC class II-positive MSCs incited significant proliferation of T cells from responder horses of all ELA haplotypes (A2, A3, and A9) examined in this study. This underscores the caution that must be taken when using allogeneic MSCs.

Thoroughbreds were used in the present study, but most breeds of horses can have multiple ELA haplotypes [30,38]. It is therefore not safe to assume that donor MSCs from any breed of horse can be used in a recipient horse of the same breed without the potential for an immune reaction.

## Conclusions

The results of this study suggest that bone marrow-derived MSCs should be immunophenotyped and confirmed as MHC class II negative before allogeneic application. As demonstrated, examination of MSCs for classic morphologic characteristics cannot be used alone to assess the potential for MHC class II expression. Although all MSCs in this study that displayed less-desirable morphologic characteristics were MHC class II positive, some MSCs displaying the classic morphology were also found to be MHC class II positive. Both the quantity and quality of MSCs decreases with advanced donor age [39-41], so younger horses are sought as donors. However, in the present study, even MSCs isolated from the younger horses that had the classic morphology were MHC class II positive. Additionally, it must be considered that even MHC class II-negative MSCs could potentially upregulate their MHC class II expression if implanted into an area of active inflammation, as was demonstrated on *in vitro* stimulation with IFN-γ.

In summary, we have shown that equine MSCs are heterogeneous in MHC class II expression and that MHC-mismatched MHC class II-positive MSCs are capable of inciting an immune reaction *in vitro*. All potential donor MSCs should therefore be immunophenotyped and screened for MHC class II expression. Further studies are warranted to determine the *in vivo* response to MHC-mismatched MSCs that are either MHC class II negative or MHC class II positive, as well as the effect of multiple injections of these cells on a recipient’s immune response. Further research is also required to determine the effect of MHC class II expression on equine MSC immunosuppressive properties.

## Abbreviations

APC: Allophycocyanin; CFSE: 5(6)-carboxyfluorescein diacetate *N*-succinimidyl ester; DF: dermal fibroblast; ELA: equine leukocyte antigen; FITC: fluorescein isothiocyanate; GMFI: geometric mean fluorescence intensity; IFN-γ: interferon-gamma; MHC: major histocompatibility complex; MSC: mesenchymal stromal cell; PBL: peripheral blood leukocyte; PBS: phosphate-buffered saline.

## Competing interests

The authors declare that they have no competing interests.

## Authors’ contributions

All authors conceived of the study and participated in the planning and coordination of experiments. LVS, DFA, and MJBF were primarily responsible for the MLR study design. LVS and LMP performed all cell isolation and characterization. LVS carried out the trilineage differentiation assays. LVS and LMP performed all MLR experiments. All authors contributed to data analysis and interpretation. LVS and LAF were primarily responsible for writing the manuscript. All authors edited the draft manuscript and read and approved the final manuscript.

## Supplementary Material

Additional file 1: Figure S1 Examples of bone marrow-derived mesenchymal stromal cell (MSC) morphology observed for MHC class II-negative cells **(A and B)** and MHC class II positive cells **(C through F)**. Note that some MHC class II negative cells **(C and D)** displayed the classic spindle-shape morphology equivalent to that observed for MHC class II negative cells, whereas others displayed a less characteristic morphology **(E and F)**. Also note that the MSCs maintained their morphology over multiple passages whether they converted to MHC class II negative or not. In the example shown here, MSCs from horse 9 had the same morphology at P2 (MHC class II positive; **D**) as they did at P5 (MHC class II negative; **B**). Click here for file

Additional file 2: Figure S2 Flow-cytometric histogram analyses of MHC class II expression in passage 2 (P2) and passage 8 (P8) bone marrow-derived mesenchymal stromal cells (MSCs). The open lines represent negative isotype control staining, and the shaded curves represent MHC class II staining. The percentage of positive cells is described in the upper right corner of each histogram. Note the variability in both the percentage of cells positive for MHC class II expression at P8 as well as the variability in fluorescence intensity for those MSCs that remained MHC class II positive at P8 (horses 7 and 10 in this figure). Click here for file

Additional file 3: Figure S3 Responder T-cell proliferation results for individual experiments **(A through E)** used to generate Figure 1A and B. MHC-M, MHC-matched; MHC-MM, MHC-mismatched. Note that for every experiment, the responder T-cell proliferation in response to MHC-mismatched MHC class II-positive MSCs was greater than that observed for the negative/baseline control of MHC-matched PBLs (MHC-M MLR), MHC-mismatched MHC class II-negative MSCs, MHC-matched MSCs. Click here for file

Additional file 4: Figure S4 Dot-plot (FSC versus SSC) of gated P2 MSCs from horse 9. These MSCs were a homogeneous population within the MSC gate but were positive for MHC class II expression and displayed a diffuse or broad MHC class II expression peak on flow-cytometry histogram analysis, as shown in Additional file 2: Figure S2. This suggests that the individual MSCs themselves varied in terms of the number of MHC class II molecules expressed on their cell surfaces. All MSCs examined displayed a similar homogeneous population within the MSC gate. Click here for file
