# Supplementary figures and images for: Equine bone marrow-derived mesenchymal stromal cells are heterogeneous in MHC class II expression and capable of inciting an immune response in vitro
Source: Stem Cell Res Ther. 2014 Jan 24;5(1):13. doi: 10.1186/scrt402 (PMC4055004; doi:10.1186/scrt402)

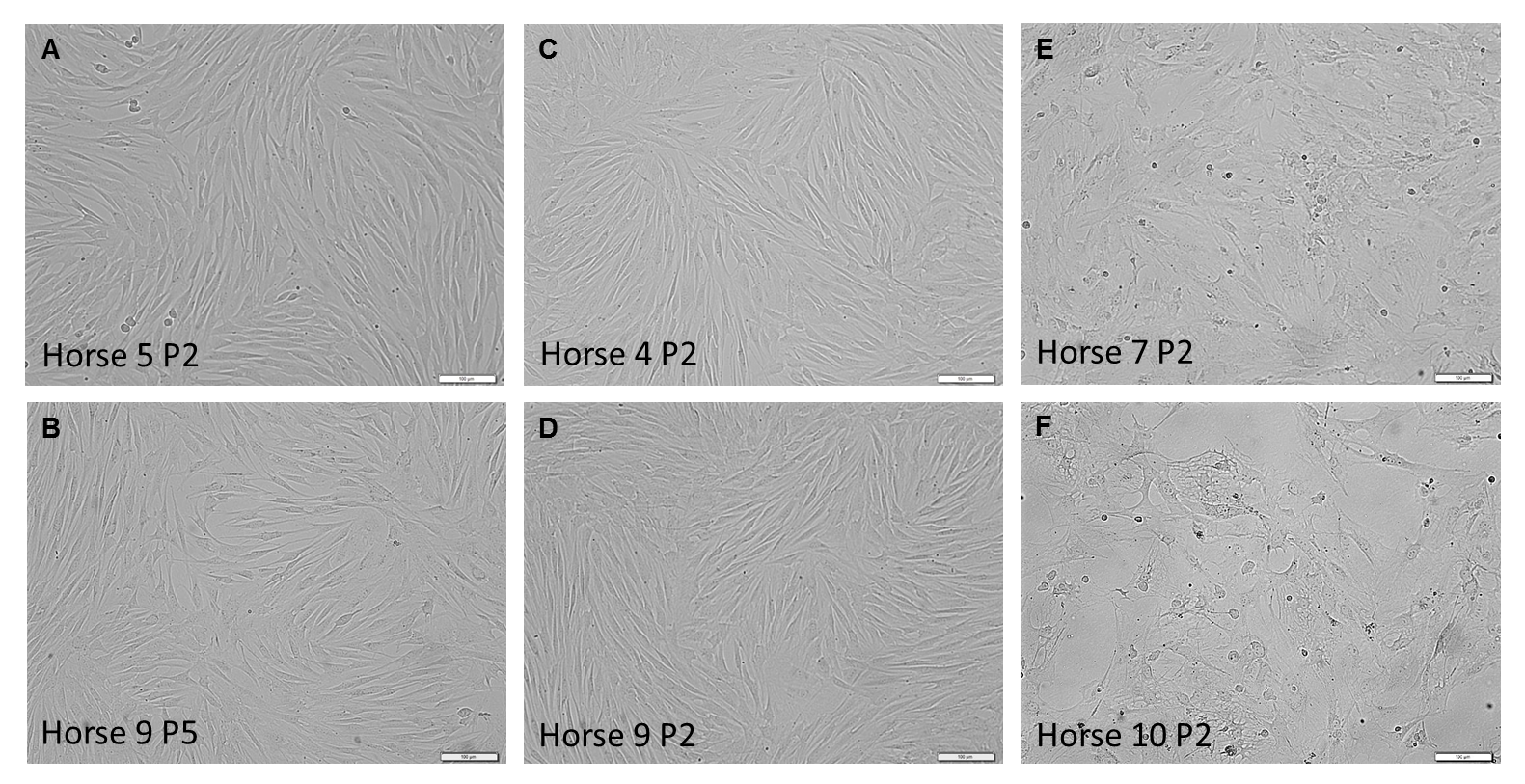

Supplement: Additional file 1: Figure S1 — Examples of bone marrow-derived mesenchymal stromal cell (MSC) morphology observed for MHC class II-negative cells (A and B) and MHC class II positive cells (C through F). Note that some MHC class II negative cells (C and D) displayed the classic spindle-shape morphology equivalent to that observed for MHC class II negative cells, whereas others displayed a less characteristic morphology (E and F). Also note that the MSCs maintained their morphology over multiple passages whether they converted to MHC class II negative or not. In the example shown here, MSCs from horse 9 had the same morphology at P2 (MHC class II positive; D) as they did at P5 (MHC class II negative; B). [file scrt402-S1.tiff]

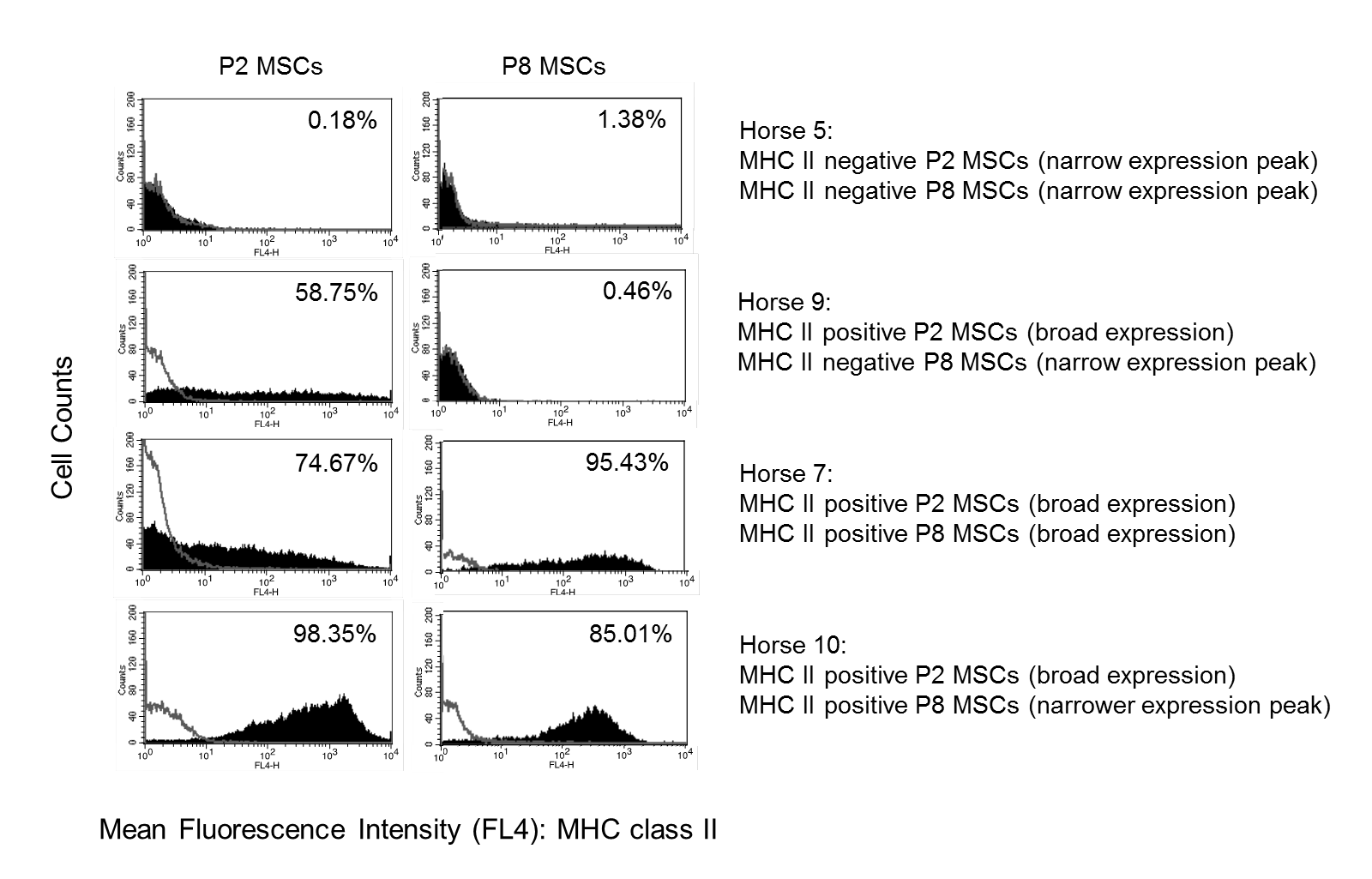

Supplement: Additional file 2: Figure S2 — Flow-cytometric histogram analyses of MHC class II expression in passage 2 (P2) and passage 8 (P8) bone marrow-derived mesenchymal stromal cells (MSCs). The open lines represent negative isotype control staining, and the shaded curves represent MHC class II staining. The percentage of positive cells is described in the upper right corner of each histogram. Note the variability in both the percentage of cells positive for MHC class II expression at P8 as well as the variability in fluorescence intensity for those MSCs that remained MHC class II positive at P8 (horses 7 and 10 in this figure). [file scrt402-S2.tiff]

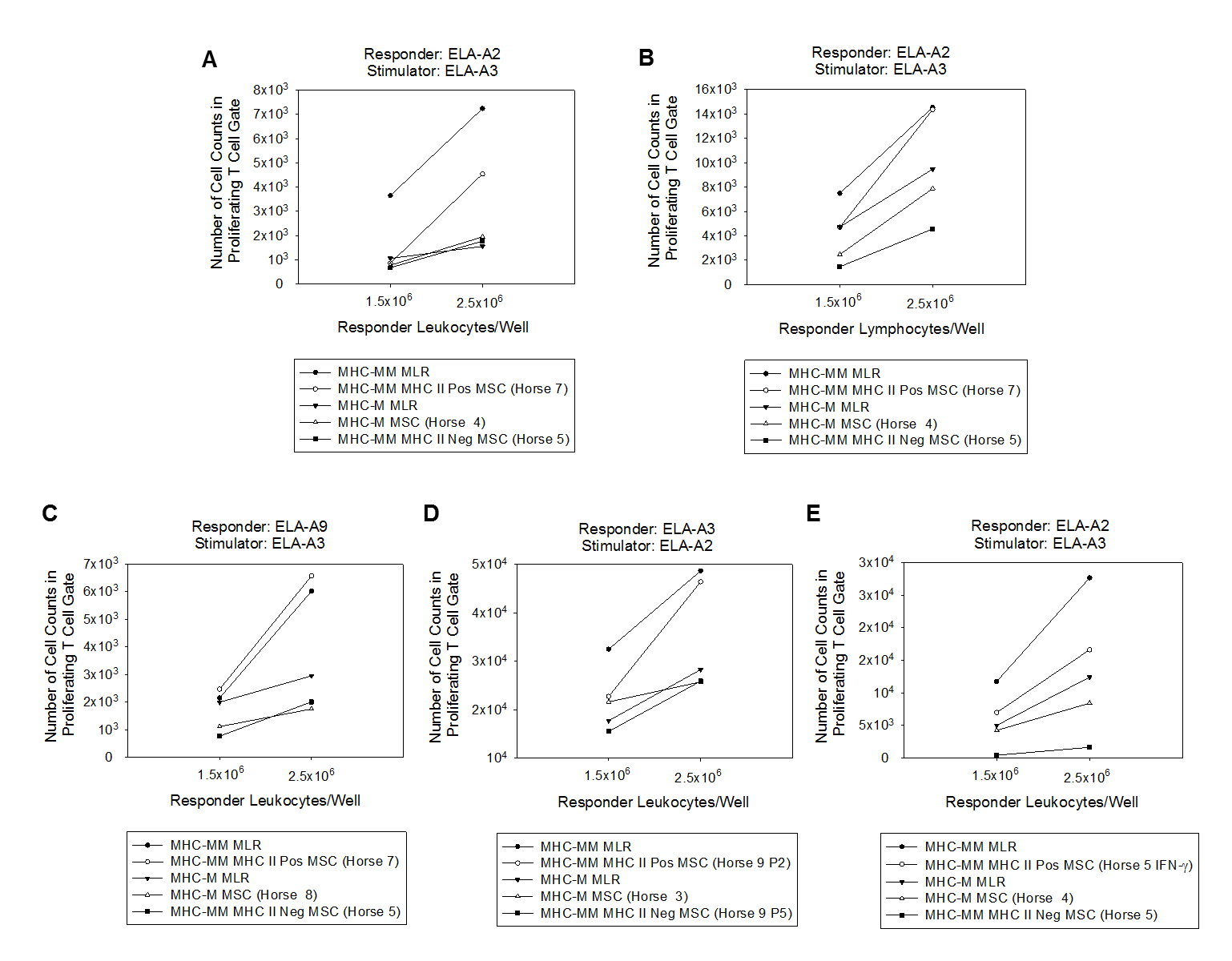

Supplement: Additional file 3: Figure S3 — Responder T-cell proliferation results for individual experiments (A through E) used to generate Figure 1A and B. MHC-M, MHC-matched; MHC-MM, MHC-mismatched. Note that for every experiment, the responder T-cell proliferation in response to MHC-mismatched MHC class II-positive MSCs was greater than that observed for the negative/baseline control of MHC-matched PBLs (MHC-M MLR), MHC-mismatched MHC class II-negative MSCs, MHC-matched MSCs. [file scrt402-S3.tiff]

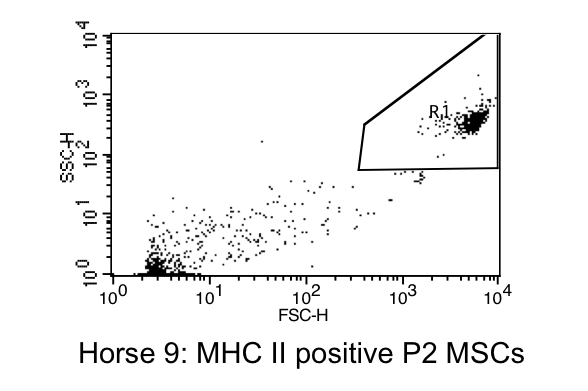

Supplement: Additional file 4: Figure S4 — Dot-plot (FSC versus SSC) of gated P2 MSCs from horse 9. These MSCs were a homogeneous population within the MSC gate but were positive for MHC class II expression and displayed a diffuse or broad MHC class II expression peak on flow-cytometry histogram analysis, as shown in Additional file 2: Figure S2. This suggests that the individual MSCs themselves varied in terms of the number of MHC class II molecules expressed on their cell surfaces. All MSCs examined displayed a similar homogeneous population within the MSC gate. [file scrt402-S4.tiff]
